# Sounds of the future and past

**DOI:** 10.1111/bjop.12753

**Published:** 2024-12-09

**Authors:** David M. Sidhu, Johanna Peetz

**Affiliations:** ^1^ Carleton University Ottawa Ontario Canada

**Keywords:** embodiment, linguistic metaphor, perceptual associations, sound symbolism, time

## Abstract

We report evidence of sound symbolism for the abstract concept of time across seven experiments (total *N* = 825). Participants associated the future and past with distinct phonemes (Experiment 1). In particular, using nearly 8000 pseudowords, we found associations between the future and high front vowels and voiced fricatives/affricatives, and between the past and /θ/ and voiced stops (Experiment 2). This association was present not only among English speakers but also by speakers of a closely related language (German) and those of a more distantly related language (Hungarian; Experiment 3). This time‐sound symbolism does not appear to be due to embodied articulation (Experiment 4). In sum, these studies identify a robust time sound symbolism effect, along with tests of underlying mechanisms.

## BACKGROUND

Sound symbolism is a phenomenon in which the sounds of language (i.e., *phonemes*) seem inherently associated with certain properties or dimensions. For instance, the invented word ‘maluma’ is associated with roundness while the word ‘takete’ is associated with spikiness (Ćwiek et al., 2022; *Köhler, W*., 1929). Something in the phonemes of either word leads to an association with round and smooth contours, or spiky and abrupt ones. Sound symbolism is of interest because it presents one way in which language can be *iconic* and form can imitate meaning. For instance, a word like ‘cactus’ contains phonemes associated with spikiness and refers to a spiky object. This stands in opposition to the dictum that the relationship between form and meaning is arbitrary in spoken language (Hockett, 1963), with no possible meaningful relationship between the two. Instead, work has shown that many words in spoken language are rated as iconic (Perry et al., 2015; Thompson et al., 2020; Winter, Perlman, et al., 2022) and that iconicity has effects on language acquisition (e.g., Perry et al., 2015; Sidhu, Williamson, et al., 2022) and processing (e.g., Sidhu et al., 2020).

Sound symbolism is not limited to shape. For instance, there has been a great deal of work demonstrating that phonemes are also associated with different *sizes* (e.g., Sapir, 1929; Thompson & Estes, 2011). High‐front vowels (e.g., /i/, as in ‘bee’) are associated with smallness, while low‐back vowels (e.g., /ɑ/, as in ‘balm’) are associated with largeness. Research has also demonstrated associations between phonemes and brightness (see Johansson et al., 2020), speed (Cuskley, 2013), taste (Gallace et al., 2011), valence (Yu et al., 2021), personality (Sidhu et al., 2019) and others (for reviews see Lockwood & Dingemanse, 2015; Sidhu & Pexman, 2018). Interestingly, these effects are not distinct, and there seem to be patterns in terms of the dimensions that are associated with a given phoneme (see Sidhu, Vigliocco, & Pexman, 2022; Tzeng et al., 2017). For instance, high‐front (low‐back) vowels are not only associated with smallness (largeness) but also brightness (darkness) and quickness (slowness). This highlights the *pluripotentiality* of phonemes (see Akita et al., 2024; Winter et al., 2019). That is, a single phoneme can afford associations with a variety of different dimensions (e.g., /i/ is both small and bright), with each being highlighted under different contexts.

Sound symbolism is often explained through imitative links between phonemes' features and a given perceptual dimension. For example, the association between /i/ (/ɑ/) and small (large) sizes can be explained through the amount of open space in the mouth as phonemes are articulated (Sapir, 1929). This space is small in the case of /i/ and large in the case of /ɑ/. This imitation can also occur crossmodally through analogy. For example, Fort and Schwartz (2022) demonstrated that continuous (discontinuous) sounds were associated with round (spiky) outlines. Thus, continuity in sound mapped onto continuity in shape.

The purpose of the present study is to test for sound symbolism beyond concrete perceptual dimensions such as shape or size, instead focusing on the abstract dimension of time. Notably, time is not defined by perceptual features that would invite imitative links with phonemes, but rather it is an abstract concept.^1^ We examine whether sound symbolism can exist for such a dimension. We next present two ways in which this might occur. In both cases, phonemes are associated with properties related to time, rather than time itself.

### Sound symbolism via perceptual associations

While time is an abstract concept and lacks perceptual features in and of itself, it is a rich mental construct that is linked to other cognitive and perceptual qualities. The future is represented in more abstract, prototypical features (Kane et al., 2012) and represented in more vivid and intense emotions (Caruso et al., 2008; Van Boven & Ashworth, 2007) than the past. Events or tasks in the future feel psychologically closer than events at an equidistant point in the past (Caruso et al., 2013; Van Boven & Caruso, 2015). In sum, the past and the future differ in cognitive representation, attention, and emotional associations which might contribute to time sound symbolism (see Ramos et al., 2022 for a review).

Importantly, the future and/or past may be associated with perceptual features, which could afford sound symbolic associations. For instance, the past may be associated with qualities such as darkness or slowness, while the future could be associated with qualities such as brightness^2^ or quickness. We verify these associations in a pilot study that asks participants provide associations of the ‘past’ and ‘future’. Importantly, phonemes have associations with both brightness and speed (see Cuskley, 2013; Johansson et al., 2020; Sidhu, Vigliocco, & Pexman, 2022), which could mediate association with time. Indeed, similar mediated processes have been used to explain crossmodal correspondences (see Motoki et al., 2023), as well as sound symbolism (see Akita et al., 2024; Kawahara et al., 2015; Sidhu & Pexman, 2018). Work examining sound symbolism for other non‐perceptual concepts (personality, emotion) is also consistent with such mediation via perceptual associations. For example, Kawahara et al. (2015; see also Sidhu & Pexman, 2015) showed that voiceless stops (e.g., /k/, /t/) were associated with an unapproachable personality. Their interpretation was that this was mediated by the phonemes' associations with spiky shapes (e.g., a perceptual dimension). There are similar mediated effects for emotion concepts. Yu et al. (2021; see also Körner & Rummer, 2021) demonstrated that certain phonemes (/i/, /ʌ/) are associated with positive or negative valence, respectively. They explained this effect by noting that the articulation of these vowels involves the muscles used to smile and frown, respectively (e.g., an embodied or perceptual association).

In sum, we speculate that one possible underpinning for a time sound symbolism effect might be an association of phonemes with the future and/or past via their associated cognitive, emotional and perceptual features.

### Sound symbolism via embodied conceptual metaphors

Another possibility is that phonemes could be associated with temporal direction via the conceptual metaphor that *time is space* (e.g., Gentner et al., 2002). In particular, English speakers talk about the future being ‘ahead’ and the past ‘behind’. People ‘look forward’ to the time when they can put a loss ‘behind them’. There is also evidence that these linguistic metaphors shape our mental representations of concepts. For example, Miles et al. (2010) demonstrated that English speakers moved forward while thinking about the future, and backward when thinking about the past. Thus, another possibility is that the future will be associated with articulations made towards the front of the mouth (e.g., /b/), and the past will be associated with articulations made towards the back of the mouth (e.g., /g/). Indeed, there is evidence that vowels articulated at the front or back of the mouth are associated with the forward and backward space, respectively (Vainio et al., 2023).

To illustrate the plausibility of this embodied mechanism, there is work showing that linguistic stimuli can be judged more or less positively based on the *direction* in which their component phonemes are articulated. Studies have demonstrated that pseudowords whose articulations move inwards (e.g., ‘benoka’) are rated more positively than those whose articulations move outwards (e.g., ‘kenoba’; Topolinski et al., 2014; see also Godinho et al., 2019). This is interpreted as being due to articulations replicating approach or avoidance movements.

In sum, another plausible mechanism by which sound symbolism could extend to the abstract conceptual dimension of time involves conceptual metaphors of time and space. The articulation of phonemes could embody the mental representation of the future as ahead, and the past as behind.

## PRESENT RESEARCH

The present research had several goals. We began by testing for the existence of time sound symbolism using groups of phonemes that previous work has shown to have contrasting associations in a pilot study. We then explored the perceptual and non‐perceptual associations of these phonemes (Experiment 1). Next, we conducted a large‐scale study to pinpoint the phonemes associated with the past and the future (Experiment 2) and examined whether predictions made by the resulting model would generalize to speakers of three different languages (Experiment 3). Next, we directly tested for an embodied articulatory mechanism by comparing consonants articulated at the front vs. back of the mouth (Experiment 4).

Full materials and data for each of the studies are available on OSF: https://osf.io/c4bh2/. All experiments received approval from the Carleton University Research Ethics Board. Across the Pilot Study and Experiments 1, 3 and 4, we aimed to collect samples large enough to detect effect sizes similar to those in previous sound symbolism studies using pseudoword ratings (Sidhu, Vigliocco, & Pexman, 2022) with at least 90% power. The power analysis was conducted in R using the ‘simr’ packages and the approach described in Kumle et al. (2021) and is available online at: https://osf.io/c4bh2/. In Experiment 2 we aimed to collect one rating for each of our nearly 8000 items. Table 1 presents participant demographics across all studies. All studies were conducted online.

**TABLE 1:** Descriptive statistics across experiments.

	Pilot study	Experiment 1	Experiment 2	Experiment 3	Experiment 4
Recruitment	UG Pool	UG Pool	Prolific	Prolific	UG Pool
Initial *N*	100	199	175	300	99
Exclusions	11 (failed compliance check)	2 (discontinued after 1st page)	8 (failed attention check) + 2 (not fluent in English) +2 (RT >3SD)	19 (not first/dominant language^a^) + 1 (failed attention check)	4 (failed compliance check)
Final *N*	89	197	165	280	95
Age (*M*, *SD*)	16–42 (20.36; 4.41)	17–43 (19.86; 2.77)	19–76 (40.37; 14.23)	18–72 (35.03; 11.53)	17–49 (20.19; 4.93)
Gender					
Women	73	142	81	175	70
Men	12	44	79	97	21
Nonbinary	4	6	5	3	3
Missing	‐	5	‐	4	‐
Race in %			Not collected	Not collected	
White	61.1	55.2			58.1
Black	12.2	8.3			8.6
Asian	12.2	9.4			9.7
Multi/Other	14.5	27.1			23.7
% Multilingual	70	70.7	19		67.4
% Fluent in English	100	100	100	100^a^	100
% English as Dominant Language	87.7	85	96	100^a^	91.5

*Note*: Across all demographics, percentages are calculated from available data. In each study, some participants did not report some of their demographic information (less than 1% missing data).

Abbreviations: M = Mean; RT = Reaction Time; SD = Standard Deviation; UG = Undergraduate.

^a^ For English/German/Hungarian, respectively.

## PILOT STUDY

In a pilot study, we examined whether sounds previously associated with small vs. large things are also associated with the future vs. past. This served as a proof of concept that participants could make associations between phonemes and time. We began with this contrast because these two sets of phonemes have been shown to cluster at opposite ends of a variety of dimensions (see Sidhu, Vigliocco, & Pexman, 2022). Based on previous work (see Knoeferle et al., 2017), large‐associated phonemes included back vowels (e.g., /ɑ/), sonorant consonants (e.g., /m/) and voiced stop consonants (e.g., /b/). Small‐associated phonemes included front vowels (e.g., /i/), voiceless fricatives (e.g., /f/) and voiceless stops (e.g., /p/). This pilot test included 40 pseudowords, 20 of which contained large‐associated phonemes (e.g., ‘gawb’ and ‘gobawn’) and 20 of which contained small‐associated phonemes (e.g., ‘teek’ and ‘peefik’). These pseudowords were created in pairs such that each large‐associated pseudoword had a matched small‐associated pseudoword with the same number of letters and syllables. All materials, including pseudoword stimuli, are available at the following OSF repository: https://osf.io/c4bh2/.

Participants (*N* = 89) were shown a series of trials with pseudowords and were instructed that:

Some of these words mean ‘future’ and some of these words mean ‘past’ in other languages. Please sort the words to the box you think is correct. Maybe say them out loud to get a sense of the word. We are interested in your intuition, not in the correct response, please just go with what you believe, don't look it up.

Participants responded using their computer mouse, by clicking on either a button labelled ‘means “Past”’ or a button labelled ‘means “Future”’, presented below the stimulus.

All analyses were conducted using the statistical software R (R Core Team, 2023), and the packages ‘lme4’ (Bates et al., 2015), ‘afex’ (Singmann et al., 2016), and ‘emmeans’ (Lenth et al., 2022). We always began with a model including all possible random intercepts and slopes. To deal with convergence issues and singular fits we attempted these remedies, in the following order: increasing the number of iterations, switching to the ‘bobyqa’ optimizer, removing the correlation between the random subject intercept and slope, individually removing the random slopes (beginning with the one with the lowest associated variance), and individually removing the random intercepts (beginning with the one with the lowest associated variance; see Brauer & Curtin, 2018; Meteyard & Davies, 2020).

Our analysis consisted of a mixed effects logistic regression with the likelihood that a given pseudoword was categorized as meaning the future as the dependent variable. Effects coded pseudoword type (i.e., small‐ [.5] vs. large‐associated [−.5]) was our predictor of interest. This revealed that participants were more likely to select a pseudoword with small‐associated phonemes as relating to the future as compared to a pseudoword with large‐associated phonemes (*b* = 0.52, *p* < .001). Thus, the initial pilot test suggested that temporal direction might be associated with a set of phonemes: pseudowords containing front vowels, voiceless stops and voiceless fricatives were associated with the future; while pseudowords containing back vowels, voiced stops and sonorants were associated with the past.^3^

Additionally, participants were asked to describe in open‐ended format the associations they have with the word ‘future’ and the word ‘past’, respectively. Participants' responses were coded for the associations they listed, with the most frequent associations being age (general pattern for 'future' vs. 'past': young/youth vs. old; 45%), movement (approaching vs. receding; 24%), location (ahead/in front vs. behind/after; 10%), brightness (bright vs. dark; 10%), arousal (exciting vs. calm; 10%), and openness (open vs. closed; 10%), and less common associations being speed (fast vs. slow, 6%), size (small vs. big; 7%), and valence (happy vs. sad; 4%).

## EXPERIMENT 1

In the next experiment, we aimed to replicate the temporal direction association with small‐ and large‐associated phonemes in a new sample of participants. In addition, participants rated several possible associations of the pseudowords, to examine if any of these dimensions mediated time sound symbolism. Specifically, participants rated all the dimensions that emerged as spontaneous associations in the pilot study.

### Method

The experiment was run using the experiment platform Qualtrics (https://www.qualtrics.com/). Participants (*N* = 197) were presented with 40 trials of rating pseudowords on several dimensions. This experiment used the same 40 pseudowords as the Pilot Study. Participants were instructed that:

In this study, you will be shown 40 invented words one at a time. We want you to rate these pseudowords based on the impression that you get from them. So, even though they don't mean anything, rate them based on the general impression you get from them. Importantly, some of these ratings will not be very literal. For instance, imagine that you were asked to rate a pseudoword on a scale from warm to cold. This would be difficult to do literally. However, you would be able to rate the pseudoword based on whether its sound gives off a warm or a cold impression. Feel free to use any point along the entire scale. There are no right or wrong answers; we are interested in what you feel to be the best answer. Don't spend too long on any particular rating; try to go with your first instinct.

Each trial included a single pseudoword presented in the middle of the screen in 22 pt. font. Each pseudoword was rated on several dimensions using 7‐point bipolar scales. To reduce participants' fatigue, each participant rated only five out of the 10 dimensions, though every participant rated the ‘time’ dimension. The order of presentation of the dimensions was randomized. Participants rated pseudowords on the dimensions size (*small* to *big*), speed (*slow* to *fast*), brightness (*dark* to *bright*), valence (*sad* to *happy*), arousal (*calm* to *exciting*), or the dimensions age (*young* to *old*), location (*behind* to *in front*), accessibility (*open* to *closed*), movement (*approaching* to *receding*), and everyone rated the time dimension (*past* to *future*). Participants responded using their computer mouse, by clicking on a number between 1 and 7 for each dimension.

After the association judgements, participants also completed a time perspective scale (Lang & Carstensen, 2002), which assesses an orientation towards the future (10 items, e.g., ‘My future is filled with possibilities.’, ‘I have the sense time is running out.’) on a scale from *Very untrue* (1) to *Very true* (7). The items were later aggregated for analysis (α = .85). Participants were also asked the extent to which they mouthed each word before rating it (‘When sorting these words, how often did you shape the words with your mouth before making your decision?’) on a scale from *Never/Not once* (1) to *All of the time/For all of them* (7). Finally, participants completed a brief demographic questionnaire (reporting age, gender, race, and languages spoken).

### Results

**FIGURE 1:**
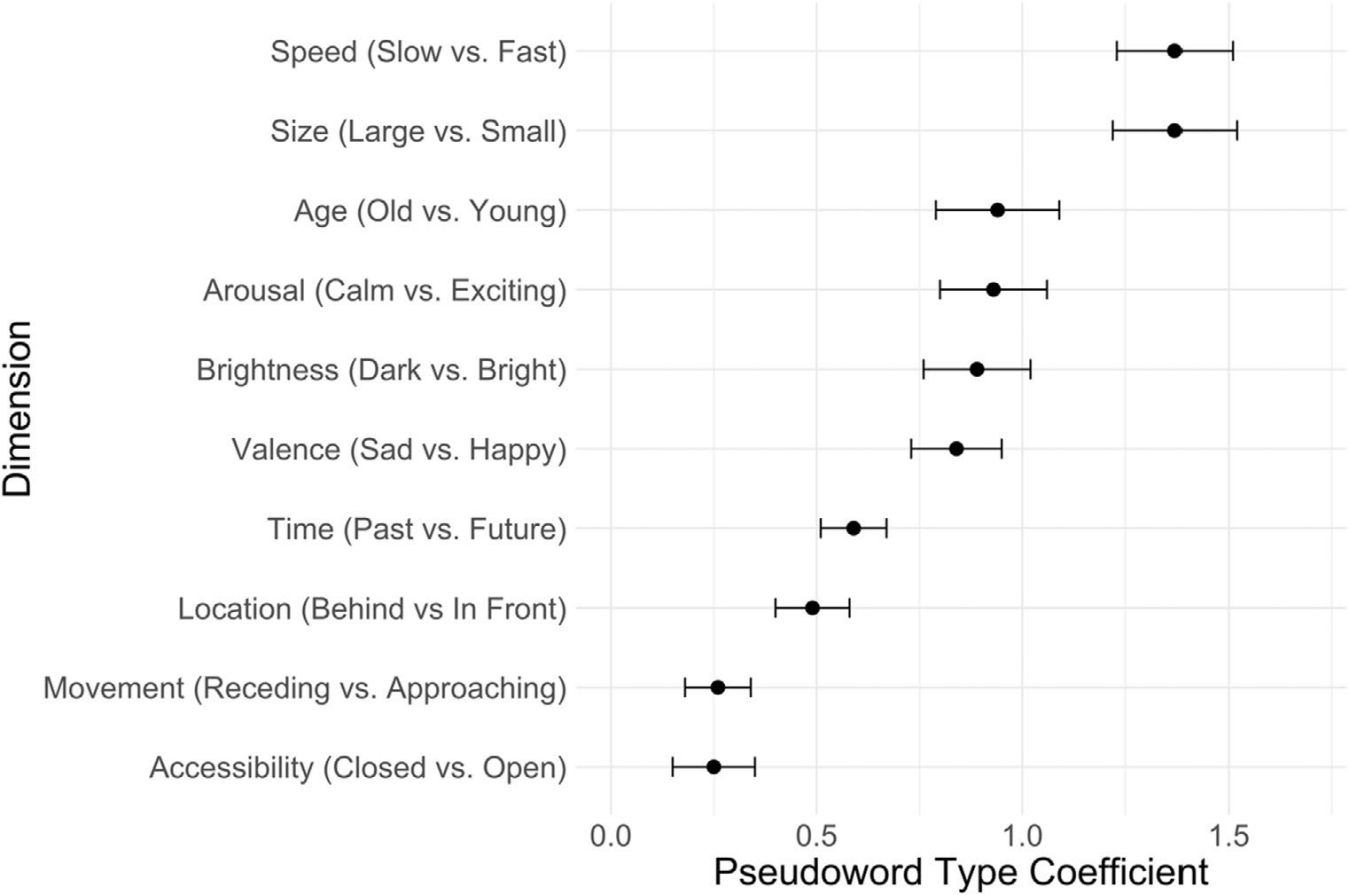
Coefficients of the relationship between word type (future‐ or past‐associated) and each of the dimensions studied. Absolute values of coefficients are shown. Values indicate the extent to which small‐ (large‐) associated pseudowords were associated with the second (first) term. The directions of the relationships were such that future‐associated pseudowords were rated as being quicker, smaller, younger, more exciting, brighter, happier, in front, approaching, and open. The order of anchors for *Small‐Big*, *Approaching‐Receding*, *Old‐Young* and *Closed‐Open* were reversed for the plot to have the future‐associated end of each dimension come second.

We began by examining on which scales future‐ and past‐associated pseudowords differed. To that end, we computed separate mixed effects linear regressions for each dependent variable scale. Our predictor of interest was effects coded pseudoword type (i.e., future [.5] vs. past [−.5]). This revealed that future‐associated pseudowords (vs. past‐associated pseudowords) were rated as being more related to the future (*b* = 0.59, *p* < .001), smaller (*b* = 1.37, *p* < .001), more exciting (*b* = 0.93, *p* < .001), brighter (*b* = 0.89, *p* < .001), happier (*b* = 0.84, *p* < .001), faster (*b* = 1.36, *p* < .001), younger (*b* = 0.94, *p* < .001), in front (*b* = 0.49, *p* < .001), open (*b* = 0.25, *p* = .02), and approaching (*b* = 0.26, *p* = .003). See Figure 1.

**FIGURE 2:**
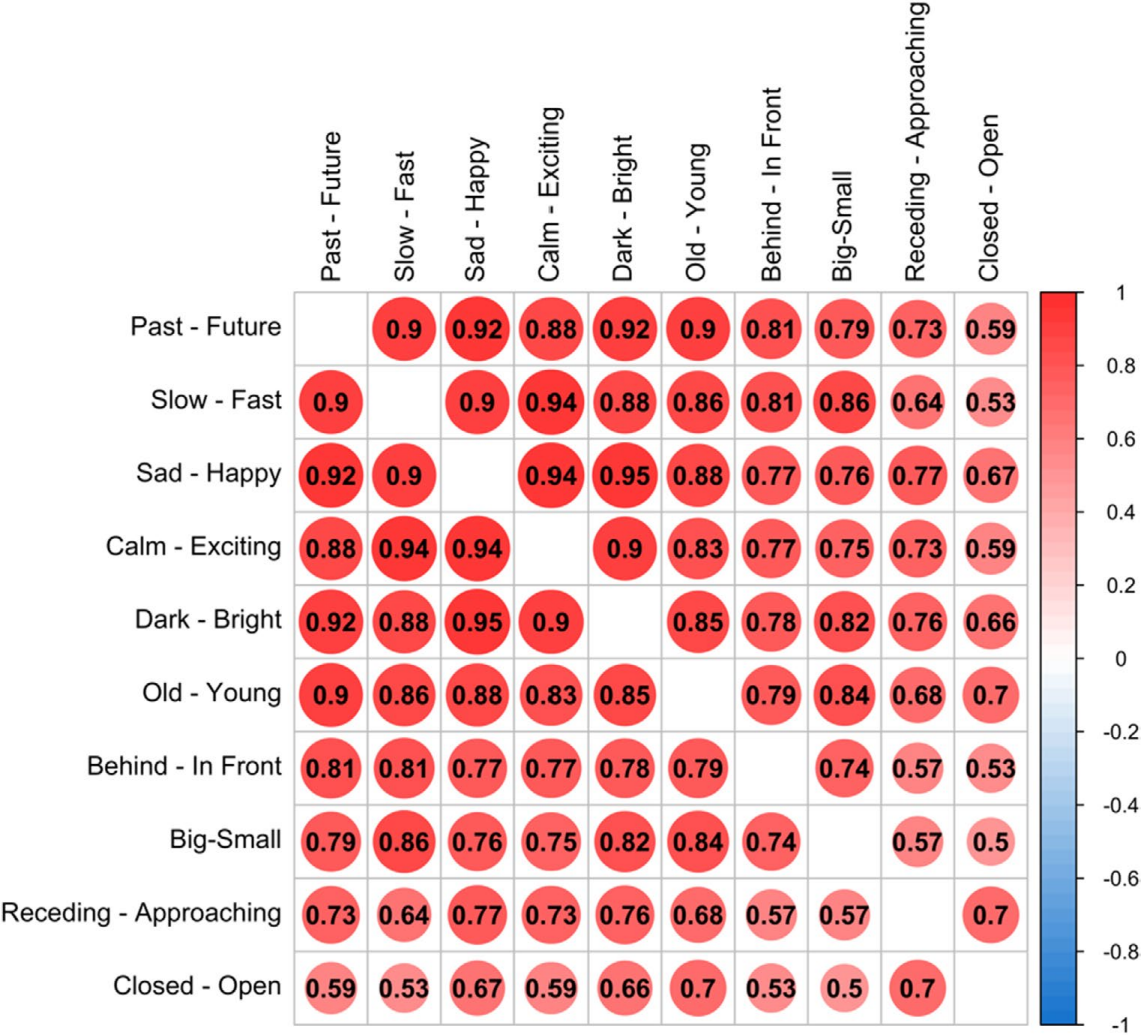
Correlations between ratings of words on each dimension. The order of anchors for *Small‐Big*, *Approaching‐Receding*, *Old‐Young* and *Closed‐Open* were reversed for the plot to have the future‐associated end of each dimension come second. Dimensions are in descending order of mediation strength.

We next used the ‘mediation’ package in R to examine which of these dimensions mediated the relationship between pseudoword type and time. These analyses revealed that each dimension (except for size) was a significant mediator at *p* < .001. However, examining the proportion of the effect which was mediated revealed that some dimensions did this to a greater extent than others. The dimensions emerging as largest mediators were speed (Proportion Mediated = 0.45), valence (Proportion Mediated = 0.40), arousal (Proportion Mediated = 0.40), brightness (Proportion Mediated = 0.31), age (Proportion Mediated = 0.27) and location (Proportion Mediated = 0.18). See Figure 2 for correlations among dimensions.

In supplementary analyses, we investigated whether ratings on any of these dimensions interacted with temporal focus scale in the prediction of ratings on the time dimension, using separate linear mixed effects models for each dimension. The interaction term was not significant in any of these models (*p*'s > .07). A supplementary analysis tested an interaction between future‐ vs. past‐associated pseudowords and the extent to which participants reported mouthing words during the task, in the prediction of ratings on the time dimension. This interaction was not significant (*b* = 0.01, *p* = .92).

### Discussion

This experiment replicated the association of the future and the past with small‐ and large‐associated phonemes, respectively. It also highlights the pluripotentiality of phonemes: each phoneme was associated with multiple dimensions. Notably, the results also suggested that a variety of dimensions previously shown to be implicated in sound symbolism (Sidhu, Vigliocco, & Pexman, 2022), and spontaneously associated with temporal dimensions in the pilot study, mediated time sound symbolism. Chief among these were: speed, valence, arousal, brightness, age, and location.

## EXPERIMENT 2

After the initial studies suggesting the existence of time sound symbolism, we turned to an investigation of the exact phonemes that play a role in the effect. The previous experiment used a carefully chosen set of maximally contrasting pseudowords. In the next study, we collected largescale data across thousands of pseudowords in order to more precisely characterize the phonemes involved in the effect. This study also added an auditory component, presenting pseudowords both auditorily and visually.

### Method

The experiment was conducted using the experiment platform Gorilla (https://gorilla.sc/). Participants (*N* = 165) were recruited from Prolific Academic. Stimuli were 7998 pseudoword recordings taken from Westbury et al. (2018; e.g., ‘adelous’, ‘conctic’, ‘goonx’, ‘cruckwic’). These pseudowords were created pseudo randomly in order to resemble the distributions of phonemes and phoneme combinations in English. They include 41 different English phonemes and were all three to eight letters in length. Because audio recordings were readily available for these items, and to ensure their pronunciation matched their transcriptions, stimuli were presented auditorily as well as visually. Each participant rated one of 160 randomly created lists of 50 pseudowords.

Each trial began with a 500 millisecond blank screen. Then a pseudoword appeared in the centre of the screen in size 24 font, at the same time as an audio recording of the pseudoword played. Recordings were created using Apple's text‐to‐speech software. After 3000 milliseconds (longer than any of the audio files) the pseudoword disappeared and was replaced by a rating screen. On the rating screen, participants were asked to ‘Please rate the word's association from 1 (Associated with the Past) to 7 (Associated with the Future)’. Participants responded using their keyboard, by typing a number between 1 and 7. Their response triggered a screen asking them to press the spacebar when they were ready to continue.

Each participant's final trial was always an attention check that resembled a real trial except that instead of pseudoword audio, they heard a message asking them to press 0. Finally, participants completed a brief demographic questionnaire (reporting age, gender, languages spoken and English fluency).

### Results

We conducted an analysis in which pseudowords were dummy coded based on the phonemes and phoneme categories they contained (Sidhu et al., 2021; Westbury et al., 2018). That is, for a given pseudoword, each phoneme predictor was coded as 1 if that phoneme was present in the pseudoword, and 0 if it was not. We included predictors for all possible phonemes as well as phoneme categories, see Table 2. The only exception is that the phoneme /ð/ (the consonant in 'the') only appeared in four stimuli and so it was removed as a predictor. Note that /ʊ/ (the vowel in 'hood') did not appear in any of the stimuli.

**TABLE 2:** Predictors which were entered into the best subsets regression model.

Predictor type	Predictors
Individual Phonemes	/i/, /ɪ/, /eɪ/, /ɛ/, /æ/, /aɪ/, /aʊ/, /ʌ/, /ə/, /u/, /oʊ/, /ɔ/, /ɔɪ/, /ɑ/, /p/, /b/, /m/, /w/, /f/, /v/, /t/, /d/, /tʃ/, /dʒ/, /n/, /r/, /θ/, /s/, /z/, /ʃ/, /ʒ/, /l/, /j/, /k/, /g/, /ŋ/, /h/
Phoneme Categories	High Front Vowel (monopthong unless otherwise stated), Low Front Vowel, High Back Vowel, Low Back Vowel, Front Vowel Dipthongs, Back Vowel Dipthongs, Voiceless Stop, Voiced Stop, Nasal Sonorant, Voiceless Fricative or Affricate, Voiced Fricative or Affricate, Approximant Sonorant

*Note*: Diphthongs were categorized as front or back depending on where they began.

The dependent variable was each word's rating, standardized within each participant. The analysis consisted of a best subsets regression. This analysis computes every possible combination of predictors, to determine the best model (based on sum of squared residuals) at each number of predictors. We then chose to interpret the model with the lowest Bayesian Information Criterion (BIC)—in this case the model with six predictors. This model revealed that the presence of /aɪ/ (*b* = 0.26), /ə/ (0.12), a high front vowel (0.13) and a voiced fricative/affricate (0.13) was predictive of a pseudoword being associated with the future; and that the presence of /θ/ (−0.26) and a voiced stop (−0.09) was predictive of a pseudoword being associated with the past. See Table 3 for the resulting model.

**TABLE 3:** Resulting model from best subsets regression analysis predicting pseudoword time rating on past‐future association scale.

Predictor	*B*	*SE*	*p*
/aɪ/	0.25	0.06	<.001
Voiced Fricatives and Affricates	0.13	0.03	<.001
High Front Monophthong Vowels	0.13	0.02	<.001
/ə/	0.12	0.02	<.001
Voiced Stops	−0.08	0.02	<.001
/θ/	−0.26	0.07	<.001

*Note*: Positive coefficients indicate an association with the future. /aɪ/ as in ‘high’; /ə/ is the final sound in ‘Johanna’; /θ/ is the first sound in ‘think.

Eight of the participants reported a language other than English as being their dominant language. We ran a supplementary analysis which removed these participants and found that the model with seven predictors had the lowest BIC. This model includes the same predictors as above, along with /h/ (−0.12) as an additional predictor of a pseudoword being associated with the past.

We conducted a supplementary analysis which coded consonants based on their place of articulation (ordered from front to back: bilabial, labiodental, alveolar, palatal, velar and glottal). We then ran a multiple regression including the presence of one consonant articulated at any of these locations, along with vowels (monophthongs: high‐front, low‐front, high‐back, low‐back; diphthongs: front, back) as predictors, see Table 4. This model suggested that bilabials (*b* = 0.06, *p* = .009), labiodentals (*b* = 0.07, *p* = .013) and alveolars (*b* = 0.08, *p* = 024) are associated with the future while the glottal phoneme /h/ (the only glottal phoneme) is associated with the past (*b* = −0.09, *p* = .049). This is consistent with an explanation based on articulation; however, we are hesitant to overinterpret this exploratory analysis.

**TABLE 4:** Resulting model from multiple regression including place of consonant place of articulation and vowel type predicting pseudoword time rating on past‐future association scale.

Predictor	*B*	*SE*	*p*
Bilabial	0.06	0.02	.009**
Labiodental	0.07	0.03	.013**
Alveolar	0.08	0.03	.024*
Palatal	0.00	0.02	.888
Velar	−0.03	0.02	.186
Glottal	−0.09	0.05	.049*
High‐Front Vowel	0.13	0.02	<.001***
High‐Back Vowel	0.09	0.04	.032*
Low‐Front Vowel	0.07	0.03	.011**
Low‐Back Vowel	0.00	0.06	.981
Front Diphthong	0.13	0.04	.001**
Back Diphthong	0.04	0.04	.283

*Note*: Consonants are ordered from a place of articulation at the front of the mouth (top) to the back (bottom).

* *p* < .05.

** *p* < .01.

*** *p* < .001.

### Discussion

This large‐scale study of time sound symbolism supported many of the findings from previous studies. Notably, while previous studies used a limited set of carefully chosen pseudowords, the present study found that time sound symbolism emerges across a diverse set of nearly 8000 pseudowords. In particular, high front vowels, and the front dipthong /aɪ/ were associated with the future, while voiced stops were associated with the past. We also observed the novel finding that voiced fricatives (i.e., /z/, /v/ and /ʒ/) and affricates (i.e., /dʒ/) were associated with the future, and the phoneme /θ/ was associated with the past. In the next study, we aimed to validate these findings by testing predictions from this model in speakers of three languages.

## EXPERIMENT 3

In this experiment, we validated the time associations identified in Experiment 2 in speakers of three different languages. In addition to testing the predictions made by the model built in Experiment 2, this tested the possibility that the effects observed thus far are limited to English speakers, perhaps due to patterns in the English language (i.e., systematicity; see Akita & Imai, 2022; Dingemanse et al., 2015). Thus, as a test of the effect's generalization, we tested German and Hungarian speakers. German is of the same language family as English (Harbert, 2006), and thus a sample of German speakers represents a close replication sample to the previous samples. Unlike English and German, Hungarian is not an Indo‐European language; instead, it belongs to the Uralic language family, and thus a sample of Hungarian speakers represents a more distant replication sample (for a similar use of Hungarian see Winter, Sóskuthy, et al., 2022). Quantifying these languages' relative similarity to English, the lexical similarity between German and English is 4.72 (calculated using the similarity of cognates), and the similarity between English and Hungarian is 1.84, and (Bella et al., 2021).

Specifically, using the international crowdsourcing platform Prolific Academic (https://www.prolific.com/), we recruited 100 participants living in the US who spoke English as first language and as primary/frequently spoken language, 100 participants living in Germany who spoke German as first language and as primary/frequently spoken language, and 100 participants living in Hungary who spoke Hungarian as first language and as primary/frequently spoken language.

### Method

Pseudoword stimuli consisted of 20 pseudoword recordings chosen from the stimuli in Experiment 2. In particular, we used the model created in Experiment 2 to predict each nonword's time association, ranging from strongly past‐associated to strongly future‐associated. We selected stimuli in pairs. Each pair contained a word with a score at either extreme and had the same number of letters and syllables. In addition, because many past‐associated pseudowords contained the phoneme /θ/, we purposefully chose four out of ten that did not contain this phoneme to increase the variety of sounds used. We also excluded pseudowords that we knew to be words in German or Hungarian. This resulted in the following ten future‐associated pseudowords: *cyzher, knige, hysirt, vite, xylage, fize, zylort, wrige, frisort, clige*; and the following ten past‐associated pseudowords: *utthur, galth, sothlo, crog, bothet, plob, uthoog, bulth, bongard, glonk*.

The experiment was run using the platform Qualtrics. The survey language was matched to the participant's language (i.e., English, German, or Hungarian). Participants (*N* = 280) were presented with 20 trials. Each trial included one psuedoword presented in the middle of the screen in 24 pt. font along with an audio recording of the pseudoword. Participants were asked to play the audio file and determine whether they thought the word associated with the past or the future by selecting one of two buttons labelled ‘past’ and ‘future’. Finally, participants were asked to report compliance (whether they listened to all, some, or none of the audio files) and to report demographics (age, gender, first language, dominant language).

### Results

**FIGURE 3:**
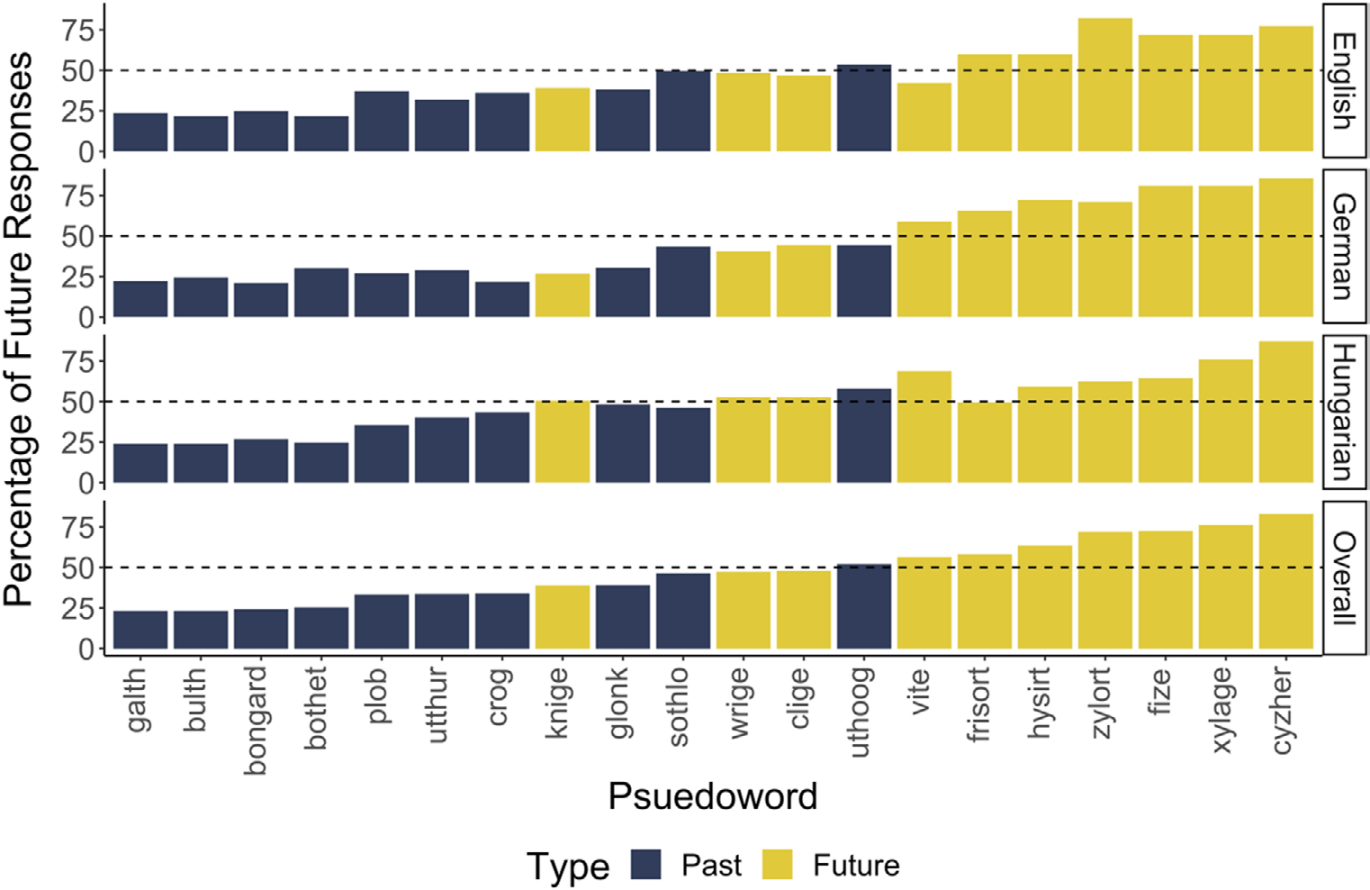
Proportion of trials on which each word was associated with the future, by pseudoword type and language group. Pseudoword order on *x*‐axis is based on the percentage of future responses for each pseudoword overall (across all language groups).

Our analysis consisted of a mixed effects logistic regression with the likelihood that a given pseudoword was categorized as meaning the future as the dependent variable. Effects coded pseudoword type (i.e., future‐ [.5] vs. past‐associated [−.5]) was our predictor of interest. This analysis also included a random effect of language. Results confirmed that across all three samples, participants were more likely to select a future‐associated pseudoword as relating to the future as compared to a past‐associated pseudoword (*b* = 1.23, *p* < .001). See Figure 3.

Next, we conducted an analysis which included dummy coded language (with English as the reference level) as a fixed effect, in addition to its interaction with pseudoword type. This analysis revealed a significant interaction between language and pseudoword type for German (vs. English; *b* = 0.34, *p* = .02), but not Hungarian (vs. English; *b* = 0.04, *p* = .76). We followed up on the significant interaction using estimated marginal means for the effect of pseudoword type in English and German speakers separately. This revealed a significant effect of pseudoword type in both groups, however one that was larger for German speakers (*b* = 1.48, *p* < .001) compared to English speakers (*b* = 1.14, *p* < .001), meaning that German speakers were even more likely to select a future‐associated pseudoword as relating to the future as compared to a past‐associated pseudoword.

### Discussion

The results of this experiment validate the predictions of the model constructed in Experiment 2 using a new set of participants. While each pseudoword was rated once in Experiment 2, the predicted phoneme associations generated from those ratings accurately identified pseudowords that were strongly associated with the future or the past, based on responses from new group of participants. Notably, these associations generalized beyond English speakers to German and Hungarian speakers. This suggests that the time sound symbolism effects identified here are not limited to speakers of a single language.

## EXPERIMENT 4

In the next experiment, we tested a possible embodied explanation for time sound symbolism. In particular, we examined whether phonemes articulated at the front vs. back of the mouth could be associated with the future vs. past, via the conceptual metaphor that the past is in front, and the past is behind. There are several factors that make this explanation worth investigating. First, one of the most common associations that pilot participants provided for time was location (i.e., future in front; past behind). Second, in Experiment 1, location ratings mediated a considerable portion of the variance in time ratings. Finally, the supplementary analysis in Experiment 2 which coded consonants based on their front to back place of articulation was consistent with this explanation.

To test an embodied explanation for time sound symbolism, we directly manipulated consonant place of articulation (back vs. front of the mouth) in a set of pseudowords. We focus on consonants because front and back vowels have been shown to have strong associations with various dimensions (see Lockwood & Dingemanse, 2015; Sidhu, Vigliocco, & Pexman, 2022), which might interfere with a test of the role of articulation, and because of the unexpected future associations of high back vowels in Experiment 2. On the other hand, consonants articulated at the back vs. front of the mouth have not been shown to have systematically different associations.^4^

### Method

Participants were recruited from an undergraduate student pool (*N* = 95). The procedure was identical to Experiment 1 but included 24 pseudowords that contained consonants articulated in the back vs. the front of the mouth. Of these, 12 were critical pseudowords while 12 were fillers. The 12 critical stimuli included six with a velar consonant (/g/ or /k/; articulated at the back of the mouth) and six with a bilabial consonant (/b/ or /p/; articulated at the front of the mouth). These pseudowords contained the vowels /ɪ/, /ʌ/, /ɛ/ or /æ/. Stimuli were yoked such that each velar pseudoword had a matched bilabial pseudoword with the same vowel and consonant voicing (e.g., /kɪg/ was matched with /pɪb/). The 12 filler pseudowords contained a variety of consonants to obscure the critical manipulation. We only analysed trials including our 12 critical items.

As in the Pilot Study and Experiment 1, stimuli were presented visually. Participants instructed that:

Some of these words could mean ‘future’ and some of these words could mean ‘past’ in other languages. Please sort the words to the box you think is correct. Maybe say them out loud to get a sense of the word. We are interested in your intuition, not in the correct response, please just go with what you believe, don't look it up.

We presented words visually, with the suggestion that participants say them out loud, to allow for the greatest chance of articulation having an effect.

Participants were also asked the extent to which they mouthed each word before rating it (‘When sorting these words, how often did you shape the words with your mouth before making your decision?’) on a scale from ‘Never/Not once’ (1) to ‘All of the time/For all of them’ (7).

### Results

Our analysis consisted of a mixed effects logistic regression with the likelihood that a given pseudoword was categorized as meaning the future as the dependent variable. Effects coded consonant location (i.e., front [.5] vs. back [−.5]) was our predictor of interest. This analysis revealed a trend in which participants were marginally more likely to associate pseudowords containing consonants articulated at the *back* of the mouth with the future (*b* = −0.30, *p* = .09) rather than the past.

A supplementary analysis tested an interaction between articulation location and the extent to which participants reported mouthing words during the task. This interaction was not significant (*b* = 0.07; *p* = .64).

### Discussion

The results of the current experiment were not consistent with embodied place of articulation as a mechanism for time sound symbolism. In fact, the results trended in the opposite direction of what one would expect. Taken together with the lack of interaction of degree to which words were ‘mouthed’ during the task (which should increase embodiment effects if any exist), it appears unlikely that embodied articulation is an explanation for the time sound symbolism effect.

## GENERAL DISCUSSION

Across multiple experiments, we consistently found evidence of sound symbolism for the abstract concept of time. Participants associated the future with small‐associated phonemes, and the past with large‐associated phonemes (Experiment 1). More specifically, high front vowels and voiced fricative/affricative were associated with the future whereas the presence of /θ/ and a voiced stop were associated with the past (Experiment 2). Pseudowords with these auditory features were perceived as more associated with the future and past, respectively, not only by English speakers but also among speakers of a closely related language (German) and those of a more distantly related language (Hungarian; Experiment 3). Thus, the tendency to associate specific phonemes with specific temporal concepts appears to be robust and replicable.

Notably, time is only one of many possible associations that people make to the sounds of language. Phonemes demonstrate pluripotentiality and possible associations with many different dimensions. It is likely that a potential association is highlighted by task instructions. Once a particular dimension becomes salient, individuals can look for possible analogies to draw between phonemes and that dimension. What we have shown is that there are consistent intuitions in terms of which phonemes should be paired with the future or the past. This demonstrates that language sounds can afford associations with the abstract dimension of time.

There are several possible explanations for this time sound symbolism, though the present studies cannot determine the exact mechanism with confidence. On the one hand, an exploratory analysis in Experiment 2 which coded consonants by place of articulation was consistent with an embodied mechanism. That is, consonants articulated at the front of the mouth were associated with the future, while the only glottal consonant (/h/), articulated at the back of the mouth, was associated with the past. On the other hand, while front vowels were associated with the future, so were high back vowels. In addition, there was no support for an embodied explanation in Experiment 4, and no evidence that time sound symbolism was moderated by the extent to which words were explicitly mouthed (Experiment 1 and 4). Instead, it appears more likely at present, that the underlying mechanism for time sound symbolism might be a series of conceptual associations (e.g., brightness, speed; Experiment 1) that link the identified phonemes to concepts of time. This explanation is consistent with work showing that there are *clusters* of sound symbolism effects (e.g., Sidhu, Vigliocco, & Pexman, 2022; Tzeng et al., 2017). In particular, Sidhu, Vigliocco, & Pexman (2022) showed that sound symbolism effects tended to cluster based on the classic semantic differential factors of valence, activity, potency, along with novelty. Time might be part of a rich nomological network of associations (also see Ramos et al., 2022) that combine to create associations between phonemes and these concepts.

Another possible contributing factor that we did not test directly is the contrasting psychological distance of the future and the past. Previous work has shown there is a tendency for the future to be perceived as psychologically closer than the past (Caruso et al., 2013; Van Boven & Caruso, 2015). This is notable because front vowels (vs. back vowels) are associated with close (vs. far) spatial distances (e.g., Rabaglia et al., 2016).

### Theoretical contributions

The main theoretical contribution of this work was to extend sound symbolism to the abstract dimension of time. The classic and most well‐studied examples of sound symbolism have involved perceptual dimensions (i.e., shape, Köhler, W., 1929; size, Sapir, 1929), with phonemes imitating these dimensions directly or through analogy. The present work suggests that the crossmodal associations of phonemes go beyond perceptual dimensions. Along with recent work demonstrating emotion sound symbolism (e.g., Aryani et al., 2018; Yu et al., 2021) and personality sound symbolism (e.g., Sidhu et al., 2019), our results serve to broaden the scope of sound symbolism.

A recent consensus paper on abstract concepts has stated that ‘the classical distinction between concrete and abstract concepts has clearly been overcome’ (Banks et al., 2023, p. 2). For instance, Banks and Connell (2023) found that the features of many abstract category members were strongly grounded in these sensorimotor modalities. This is consistent with our interpretation that phonemes are associated with time via its sensorimotor properties. This presents an intriguing route by which even those concepts which have traditionally been considered abstract can be related to phonemes in sound symbolism.

The present research also has relevance to the iconicity of language—the extent to which the forms of words imitate their meanings. Researchers have proposed that one of the reasons that language is not *more* iconic is because abstract meanings ‘resist’ iconicity (Lupyan & Winter, 2018). That is, the ability of iconicity to bring to mind a specific sensory meaning is antagonistic to abstraction. The present results are an interesting data point in this discussion and suggest that abstract concepts can be conveyed iconically through semantic relationships (see Akita et al., 2024).

The results of Experiment 2 are notable for taking a megastudy approach to sound symbolism. That is, unlike early studies on sound symbolism (and the present Experiments 1, 3 and 4) which tended to utilize carefully chosen pairs of pseudowords, there has been a shift towards extrapolating sound symbolism effects by collecting ratings of thousands of pseudowords, to avoid any particular combination of phonemes alone determining the effect (Aryani et al., 2020; Monaghan & Fletcher, 2019; Westbury et al., 2018). The results of Experiment 3 validated this approach, by showing that this model accurately predicted responses from new groups of participants, including German and Hungarian speakers. This finding fits with work showing that the patterns of shape sound symbolism demonstrated in English speakers extend across many, though not all, languages (Ćwiek et al., 2022; Styles & Gawne, 2017).

Beyond sound symbolism, these results also contribute to research which has examined metaphorical associations of time. In particular, a range of research has examined the abstract concept of time in relation to space (e.g., Callizo‐Romero et al., 2020; Starr & Srinivasan, 2021), emotions (Caruso et al., 2008; Van Boven & Ashworth, 2007) and cognitive representations of events (Caruso et al., 2013; Kane et al., 2012). Here we extend the perceptual metaphors/associations of time to *sound* as another perceptual dimension.

### Practical implications

Time sound symbolism could have a variety of practical applications. For instance, there is a great deal of evidence that the sound symbolism affects perceptions of product names (e.g., Klink, 2000). For instance, when asked which beer sounds darker, ‘gidan’ or ‘godan’, participants chose the latter. Further, Yorkston and Menon (2004) demonstrated that individuals preferred a brand whose name sound symbolically evoked desirable properties for a product. Participants preferred an ice cream named ‘frosh’ vs. ‘frish’ (with the former evoking more smoothness). Because time sound symbolism would go beyond any particular perceptual properties, it could have a wide range of applications in marketing (i.e., to any products that should be associated with the future or past). For example, attributes of modernity are particularly attractive to consumers (Blijlevens et al., 2009), making associations with the future a relevant and desirable property. Brand names for products including high front vowels and voiced fricative/affricative might convey an impression of the product being modern and avant‐garde. Indeed, there is work suggesting that certain sounds, such as greater complexity in sonic logos (Krishnan et al., 2012) or inward rather than outward articulations (Ingendahl & Vogel, 2022; Topolinski et al., 2015), can increase the appeal of a product. Sounds communicating a future orientation might similarly play a role in marketing, increasing appeal and willingness to pay. For example, future‐associated phonemes might be especially appealing in technology products, whereas past‐associated phonemes might appeal in nostalgic products.

### Limitations

The present research primarily focused on English‐speaking participants. While one experiment replicated the time sound symbolism effect among speakers of other languages (Experiment 3), these languages still overlap to some degree and speakers share many cultural similarities. Does time‐sound symbolism feature the same phonetic associations among those with different cultural‐linguistic conceptions of time? Just as the spatial properties of the concept of time may differ across cultures based on cultural metaphors (Gu et al., 2019), the specific sounds associated with the past and future might be different among speakers immersed in cultures with different time metaphors. This could be an interesting avenue for further tests of an embodied explanation. For instance, Aymara, a language spoken in the Andean highlands, refers to the past with the word for ‘front’ and the future with the word for ‘back’ (Núñez & Sweetser, 2006). It is also important to note that some cultures tend to look more towards the future than the past (Callizo‐Romero et al., 2020; Gu et al., 2019; Peetz & Wohl, 2019) which may alter the time‐sound symbolism—although note that the temporal focus tendencies of participants did not moderate the time‐sound symbolism effects in our studies so far (Experiment 1).

The present research is also limited in that it assesses sounds in isolation rather than as embedded in language context. We asked participants to evaluate pseudowords rather than full texts in order to isolate the relevant phonemes. We relied both on participants' internal voice when reading the pseudowords and also provided auditory clips of computer‐generated audio clips of the pseudowords (e.g., Experiment 2 and 3). This highly controlled experimental design might thus not generalize to more complex speech and language generated in face‐to‐face interactions, where many additional cues would also be present.

One puzzling finding was the strong association between the voiceless dental fricative /θ/ and the past. While we found strong evidence of this association, we aren't able to offer an explanation for it. However, one extremely speculative possibility is that it arises through ordinal suffix ‘‐th’, as in ‘fifth’ or ‘sixth’. This might imply that an event has finished. However, it will be up to future research to further investigate this association.

### Future directions

Recent work has demonstrated that many sound symbolism effects observed in the lab also appear in the lexicons of existing languages (e.g., Aryani et al., 2018; Sidhu et al., 2021; Winter & Perlman, 2021). Future work could take this approach with time sound symbolism, and examine words related to the future or the past within a language, or across languages. A future study could examine the sounds contained in these words across many languages and multiple types of time words (e.g., words referencing not only ‘future’ but also ‘hence’, ‘tomorrow’, and ‘upcoming’).

Future work should also examine moderators of the strength of potential impacts the time‐sound symbolism may have on choices. Just as disgust sensitivity amplifies the articulation effect in brand name preference (Ingendahl & Vogel, 2022), there might be some individual differences that amplify or attenuate any effects of time‐sound symbolism. For example, event vs. clock time orientation (Sellier & Avnet, 2014) and time orientation (Shipp et al., 2009) might matter to how strongly time‐sound symbolism affects individuals' preferences and choices.

## CONCLUSIONS

Mental time travel to consider the past and the future is one of the abilities unique to and characteristic of human thinking (Suddendorf et al., 2009). As William James (James, 2007) put it, the present is ‘no knife‐edge, but a saddle‐back, with a certain breadth of its own on which we sit perched, and from which we look in two directions into time’ (p. 605). The concepts of ‘past’ and ‘future’ are abstract but often understood in metaphors, such as spatial metaphors (Callizo‐Romero et al., 2020). In the present research, we identified another dimension of perceptual associations with these temporal concepts: sounds. We identified the phonemes consistently associated with the ‘past’ and the ‘future’, demonstrating a time sound symbolism effect.

## AUTHOR CONTRIBUTIONS

**David M. Sidhu:** Conceptualization; funding acquisition; writing – original draft; methodology; validation; visualization; writing – review and editing; software; project administration; formal analysis; data curation; resources; supervision; investigation. **Johanna Peetz:** Investigation; conceptualization; funding acquisition; writing – original draft; methodology; validation; writing – review and editing; project administration; formal analysis; software; data curation; resources; supervision.

## FUNDING INFORMATION

The research was funded in part by a grant of Natural Sciences and Engineering Research Council of Canada (#RGPIN‐2023‐05270) to the first author, and the Social Sciences and Humanities Research Council of Canada (#435‐2012‐1211) to the second author.

## CONFLICT OF INTEREST STATEMENT

The authors have no conflicts of interest to declare.

## Data Availability

Data, code and supplementary materials are available here: https://osf.io/c4bh2/.

## Footnotes

^1^
Brysbaert et al. (2014) collected ratings of words on a scale ranging from abstract (1) to concrete (5). Words relating to time are consistently rated towards the abstract end of the scale. For example, *past* has a mean rating of 1.70 (z score = −1.29) and *future* has a mean rating of 1.86 (z score = −1.13).

^2^
We thank the reviewer who pointed out that this could be due to idioms such as “the future is bright”.

^3^
While not central to the present paper, participants were also shown foreign words meaning “present”, “past” and “future” and asked to guess their meaning. Participants were not more likely than chance to categorize the meanings of real foreign time words (*b* = 0.01, *p* = .95). This is not surprising, as foreign language guessing accuracy is typically only slightly above chance, when a significant effect is found (Taylor, 1963). Further, while some sound symbolic associations have been shown to exist in real languages (e.g., Sidhu et al., 2021; Winter, Perlman, et al., 2022), there is no reason to expect every such association to be observable in real language. For example, Blasi et al. (2016) did not find evidence of sound symbolism in words for “large” across languages, despite the well‐documented existence of size sound symbolism.

^4^
There are several notable exceptions. Johansson and Zlatev (2013) found marginal evidence of a link between consonant place of articulation (i.e., back vs. front of mouth) and far/near spatial demonstrative terms (e.g., “that” and “this”) in 101 languages. Topolinski et al. (2014) found evidence that direction of articulation (i.e., inward vs. outward) was associated with approach vs. avoidance motivation.
